# Review of LINC00707: A Novel LncRNA and Promising Biomarker for Human Diseases

**DOI:** 10.3389/fcell.2022.813963

**Published:** 2022-01-26

**Authors:** Qinfan Yao, Zheng Li, Dajin Chen

**Affiliations:** ^1^ Kidney Disease Center, The First Affiliated Hospital, College of Medicine, Zhejiang University, Hangzhou, China; ^2^ Key Laboratory of Kidney Disease Prevention and Control Technology, Hangzhou, China; ^3^ National Key Clinical Department of Kidney Diseases, Institute of Nephrology, Zhejiang University, Hangzhou, China; ^4^ Zhejiang Clinical Research Center of Kidney and Urinary System Disease, Hangzhou, China

**Keywords:** LINC00707, clinical characteristic, function, mechanism, lncRNA

## Abstract

Long noncoding RNAs (lncRNAs) are a major type of noncoding RNA greater than 200 nucleotides in length involved in important regulatory processes. Abnormal expression of certain lncRNAs contributes to the pathogenesis of multiple diseases, including cancers. The lncRNA LINC00707 is located on chromosome 10p14 and is abnormally expressed in numerous disease types, and particularly in several types of cancer. High LINC00707 levels mediate a series of biological functions, including cell proliferation, apoptosis, metastasis, invasion, cell cycle arrest, inflammation, and even osteogenic differentiation. In this review, we discuss the main functions and underlying mechanisms of LINC00707 in different diseases and describe promising applications of LINC00707 in clinical settings.

## 1 Introduction

In recent years, a shift toward a growing interest in long non-coding RNAs (lncRNAs) has been evident (Ali and Grote, 2020). Applications of newly developed genomic high-throughput sequencing toolkits are constantly revealing unprecedented structures and emerging functions of lncRNAs (Qian et al., 2019).

LncRNAs are 200 nucleotides or longer. They belong to the noncoding RNA family (Kopp and Mendell, 2018; Bridges et al., 2021) and are incapable of coding proteins. Most lncRNAs can regulate the biological processes of diverse diseases by affecting target gene expression (Bhan et al., 2017; Tan et al., 2021). The common mechanisms mainly act through competitive endogenous RNA (ceRNA) to sponge microRNAs (miRNAs) or in direct combination with proteins (Ferrè et al., 2016). Further study of lncRNA-mediated processes may contribute to the diagnosis and therapy of relevant diseases (Tan et al., 2021).

Long intergenic non-protein coding RNA 707 (LINC00707), genomic location chromosome 10p14, and has been found to dysregulate many diseases (e.g., pneumonia, spinal cord injury (SCI), osteogenic differentiation, and multiple types of cancers). LINC00707 is implicated in the regulation of biological functions, including cell proliferation, cell apoptosis, cell migration and invasion, cell cycle control, inflammation, vasculogenic mimicry (VM) development, and osteogenic differentiation of human bone marrow-derived mesenchymal stem cells (HBMSCs). LINC00707 is closely associated with clinical manifestations of disease, like tumor size, stage, grade, lymphatic and distant metastasis, shorter overall survival times, and antitumor drug sensitivity. LINC00707 is considered a promising biomarker for the diagnosis, treatment, and prognosis of specific diseases. The aim of this review is to present an overview of the clinical features, biological functions, relevant mechanisms, and future clinical applications of LINC00707.

## 2 The Expression and Role of LINC00707 in Disease

LINC00707 is upregulated in disease such as lung cancer (Ma et al., 2018; Zhang et al., 2019; Shao et al., 2021), pneumonia (Zou et al., 2021), melanoma (Yang et al., 2018), colorectal cancer (CRC) (Shao et al., 2019; Zhu H. et al., 2019; Wang et al., 2020), glioma (Liu and Hu, 2020; Yu et al., 2021), clear cell renal cell carcinoma (Pang et al., 2020), breast cancer (BC) (Jiang et al., 2020; Yuan et al., 2020), osteosarcoma (Zhang et al., 2021), cervical cancer (Guo et al., 2021), bladder cancer (Gao and Ji, 2021), ovarian cancer (Guo et al., 2021), gastric cancer (Xie et al., 2019), hepatocellular carcinoma (Tu et al., 2019; Wang et al., 2019), and SCI (Zhu S. et al., 2019). LINC00707 dysregulation contributes to the process of osteogenic differentiation (Jia et al., 2019; Cai et al., 2020; Liu J. et al., 2020) of HBMSCs. Changes in LINC00707 expression levels are correlated with specific clinicopathological features (Table 1). LINC00707 has an extensive role in the activation of various biological functions via different mechanisms (Table 2).

**TABLE 1:** LINC00707 expression and clinical characteristics in diverse diseases.

Disease type	Expression	Clinical characteristics	Refs
Lung adenocarcinoma	upregulated	TNM stage, tumor size, lymphatic metastasis, poor prognosis, and cisplatin (DDP) resistance	34,486,476 29,482,190 31,788,103
Osteosarcoma	upregulated	—	34316513
Breast cancer	upregulated	—	32,432,749 32,273,767
Cervical Cancer	upregulated	—	34,258,298
Ovarian cancer	upregulated	overall survival	34,062,972
Bladder cancer	upregulated	tumor stage, grade, and shorter overall survival	34,192,702
M	upregulated	—	29,436,619
Gastric cancer	upregulated	tumor stage, tumor size, lymph node metastasis and poor prognosis	30,502,359
Glioma	upregulated	Karnofsky performance status (KPS) score, and WHO staging	33,542,193 33,107,401
Colorectal cancer	upregulated	tumor size, stage, lymphatic metastasis, distant metastasis, and poor survival	32,010,320 31,213,848 31,115,001
Hepatocellular carcinoma	upregulated	—	30,488,589 30,317,590
Clear cell renal cell carcinoma	upregulated	—	32,633,350
Pneumonia	upregulated	—	33,604,647
Spinal cord injury	upregulated	—	31,272,297
Osteogenic differentiation	upregulated	—	31,957,814 30,795,799
Osteogenic differentiation	downregulated	—	32,705,245

**TABLE 2:** Functions and mechanisms of LINC00707 in diverse diseases.

Disease type	Cell lines	Role	Functions	Related mechanisms	Refs
Lung adenocarcinoma	SPCA1, A549, DDP-resistant A549	tumor promoter	cell proliferation, cell cycle, apoptosis, migration, and invasion	Cdc42, and miR-145	34,486,476 29,482,190 31,788,103
Osteosarcoma	Saos-2, MG-63, U-2 OS, HOS, and SW-1353	tumor promoter	cell proliferation, migration, and invasion	miR-338-3p, and AHSA1	34,316,513
Breast cancer	MCF-10AT, MDA-MB-231, and MDA-MB-468	tumor promoter	cell proliferation, cell cycle, apoptosis, invasion, and migration	miR-30c, CTHRC1, AKBA, miR-206, Estrogen Receptor-α	32,432,749 32,273,767
Cervical cancer	HCC94, CaSki, MS751, HT-3, and C-33A	tumor promoter	cell proliferation, migration, and invasion	miR-382-5p, and VEGFA	34,258,298
Ovarian cancer	SKOV3	tumor promoter	—	—	34,062,972
Bladder cancer	UMUC3, and T24T	tumor promoter	cell proliferation, colony formation, apoptosis, and metastasis	miR-145, and CDCA3	34,192,702
Melanoma	—	tumor promoter	—	—	29,436,619
gastric cancer	BGC-823, and SGC-7901	tumor promoter	cell proliferation, and metastasis	HuR, and VAV3/F11R	30,502,359
Glioma	U87, and U251	tumor promoter	cells proliferation, migration, invasion, and VM formation	miR-613, HNRNPD, ZHX2, miR-651-3p, SP2, MMP2, MMP9, and VE-cadherin	33,542,193 33,107,401
Colorectal cancer	LoVo, HCT116, HT29, SW620, and SW480	tumor promoter	cell proliferation, cell cycle, invasion, and migration	miR-206, NOTCH3, TM4SF1, miR-485-5p, and FMNL2	32,010,320 31,213,848 31,115,001
Hepatocellular carcinoma	HepG2, Huh7, Hep3B, and SNU449	—	cell proliferation, cell cycle, colony formation, apoptosis, invasion, and migration	miR-206, CDK14, and ERK/JNK/AKT pathway	30,488,589 30,317,590
Clear cell renal cell carcinoma	786-O, and 769-P	tumor promoter	cell proliferation, migration, and invasion	EMT pathway	32,633,350
Pneumonia	MRC-5	—	cell viability, apoptosis, and inflammation	miR-223-5p	33,604,647
Spinal cord injury	PC-12	—	cell inflammation, and apoptosis	miR-30a-5p, and Neurod 1	31,272,297
Osteogenic differentiation	HBMSCs	the regulator of osteogenic differentiation	osteogenic differentiation	miR-103a-3p, DKK1, miR-145, LRP5, Wnt/β-catenin, miR-370-3p, and WNT2B	32,705,245 31,957,814 30,795,799

In subsequent sections, we focus on the expression level, clinicopathological features and biological functions of LINC00707 in a variety of diseases.

### 2.1 Cancer

#### 2.1.1 Lung Cancer

Lung cancer is a molecularly heterogeneous disease. The most common type is non-small cell lung cancer (NSCLC), which is diagnosed based on microscopic examination of morphological cell characteristics (Molina et al., 2008; Sankar et al., 2020). Most lung cancers in the United States are NSCLCs (prevalence, approximately 85%). The three major NSCLC subtypes are lung adenocarcinoma (LUAD), lung squamous cell carcinoma (LUSC), and lung large cell carcinoma (LULC) (Herbst et al., 2018; Zhao et al., 2020; Shao et al., 2021). Despite ever-improving clinical targeted therapies, the long-term survival rate among patients with LUAD is less than 20% (Molina et al., 2008). Therefore, studies to find new biomarkers to provide novel therapeutic targets and improve LUAD prognosis are needed.

**FIGURE 1:**
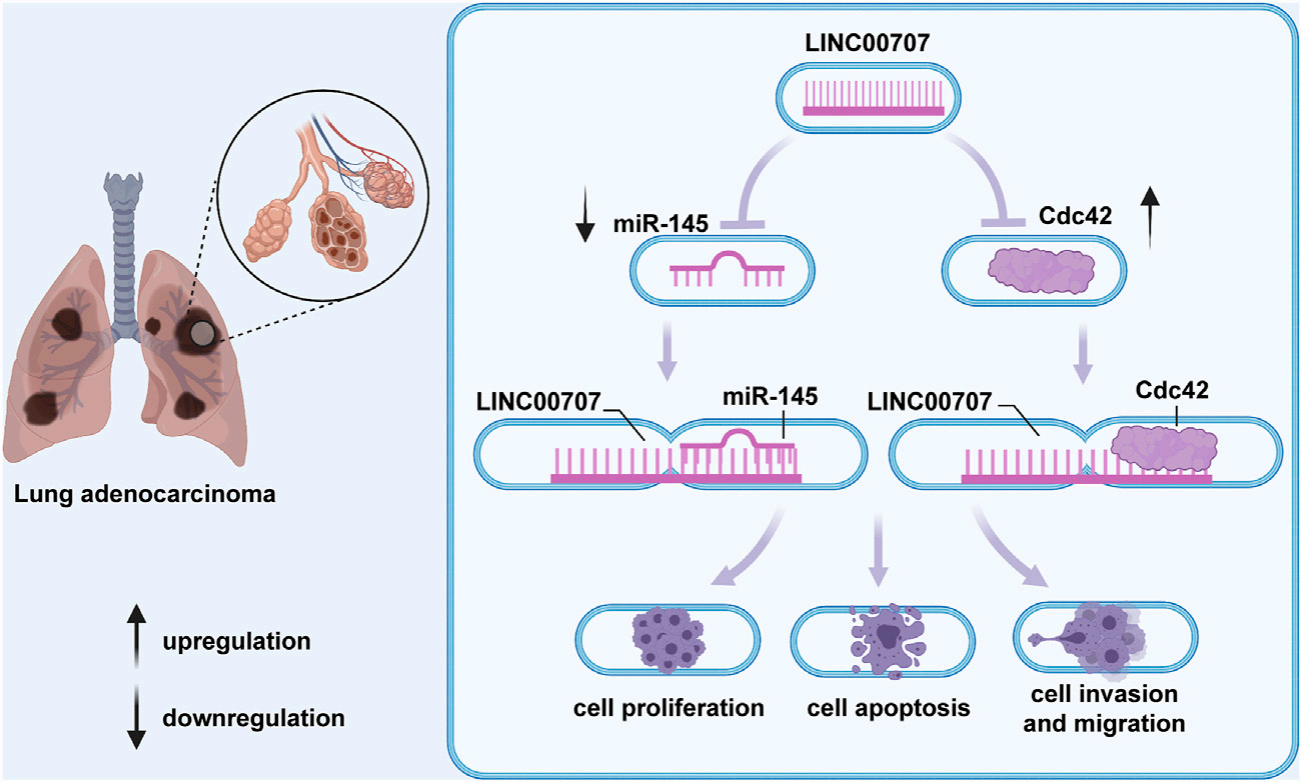
In lung adenocarcinoma, LINC00707 participates in mediation of the processes of cell proliferation, apoptosis, and migration by upregulating Cdc42 expression or combining with miR-338-3p and further increasing AHSA1 level.

Tumor hypoxia in LUAD contributes to a poor clinical outcome (Terry et al., 2020; Vito et al., 2020) by affecting the tumor immune microenvironment. LINC00707 is markedly upregulated in 64 LUAD tissues collected from First Affiliated Hospital of Nanjing Medical University and SPCA1 (Ma et al., 2018), A549, H1299, and H1975 cell lines and has been identified as a hypoxia-related lncRNA (Shao et al., 2021). High levels of LINC00707 are significantly associated with LUAD tumor size, TNM stage, distant metastasis, overall survival, and a poor prognosis (Ma et al., 2018; Shao et al., 2021). *In vitro* and *in vivo* experiments revealed that LINC00707 is involved in modulation of LUAD progression via regulation of diverse biological functions, including cell proliferation, apoptosis, and migration (Ma et al., 2018) of SPCA1 and A549 cells (Figure 1).

#### 2.1.2 Osteosarcoma

Osteosarcoma is the most common type of primary malignant bone tumor, with a peak in incidence in children, and in adolescents 15–19 years of age (Ritter and Bielack, 2010; Kansara et al., 2014). Osteosarcomas are heterogeneous tumors that lack specific targets (Corre et al., 2020) for therapy. Current combined therapeutic management approaches fail to improve overall survival of patients (Kansara et al., 2014; Corre et al., 2020; Chen et al., 2021). Genomic technologies are being applied to examine new aspects of the molecular pathogenesis of osteosarcoma to provide comprehensive insights into therapeutic strategies (Kansara et al., 2014). LINC00707 is highly expressed in osteosarcoma cell line types such as Saos-2, MG-63, U-2 OS, HOS, and SW 1353 (Zhang et al., 2021). LINC00707 can also function as an oncogene in osteosarcoma progression by regulating cell proliferation, migration, and invasion of MG-63 and Saos-2 cells (Zhang et al., 2021).

Moreover, osteogenic differentiation is the key process of development in osteosarcomas (Zhang et al., 2014; Liu et al., 2017), which has been widely explored for osteosarcomas studies. Understanding of molecular mechanisms that regulate osteogenic differentiation may hold promise for the novel treatment of human osteosarcoma (Chen et al., 2014; Modi et al., 2019; Lanzillotti et al., 2021). Recently, LINC00707 has also been found to exert an important pro-osteogenic differentiation contribution in human bone marrow mesenchymal stem cells (HBMSCs) (Jia et al., 2019; Cai et al., 2020; Liu J. et al., 2020). Most studies have found that LINC00707 expression is markedly elevated during osteogenic differentiation. The key mechanisms via which LINC00707 positively regulates osteogenic differentiation are by functioning as miR-145 (Cai et al., 2020) sponges to affect the role of LRP5 and thereby initiating the Wnt/β-catenin pathway and by sponging miR-370-3p to upregulate WNT2B (Jia et al., 2019) levels. However, one study found the opposite; LINC00707 levels were significantly decreased, and overexpression of LINC00707 repressed osteoblastic induction of HBMSCs via combination with miR-103a-3p to upregulate DKK1 expression (Jia et al., 2019).

#### 2.1.3 Breast Cancer

BC (DeSantis et al., 2011; Yin et al., 2017; Liang et al., 2020) is one of the most common female malignancies worldwide and is associated with notable incidence and mortality. Breakthroughs in the use of genomic technologies for identification and diagnosis have provided unprecedented insights into the understanding of pathogenic mechanisms of BC (Libson and Lippman, 2014; Peairs et al., 2017; Liang et al., 2020). These findings may contribute to the development of more effective systemic therapies.

**FIGURE 2:**
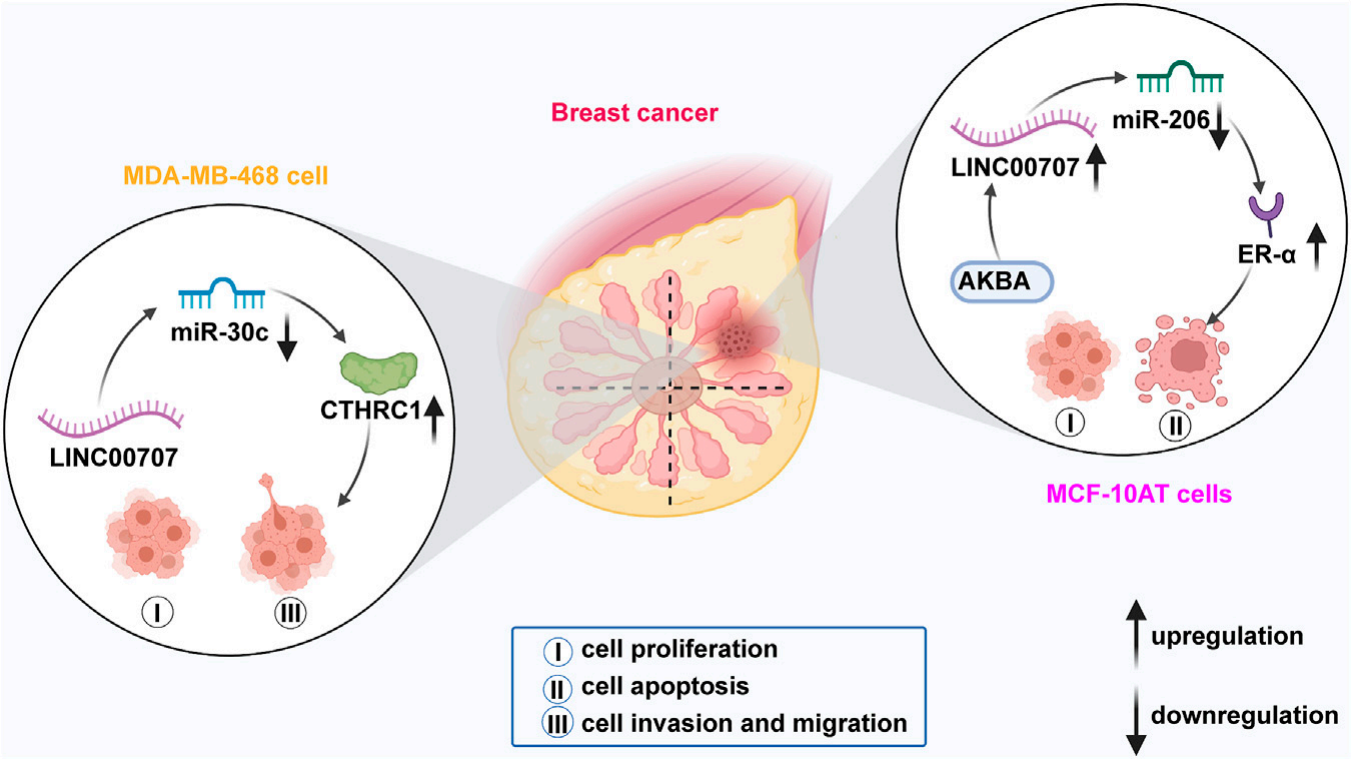
In breast cancer, LINC00707 sponges miR-30c to modulate expression of CTHRC1 and thus enhance the proliferative, invasive, and migratory abilities of MDA-MB-468 cells. LINC00707 also interacts with miR-206 to upregulate ER-α expression, which promotes the processes of proliferation and apoptosis of MCF-10AT cells.

LINC00707 is significantly upregulated in BC tissues, cell lines (MDA-MB-468, SK-BR-3, MDA-MB-415, Hs 362.T, and MDA-MB-231 cells), and precancerous cells (MCF-10AT cells) (Jiang et al., 2020; Yuan et al., 2020). LINC00707 has roles in carcinogenesis in cell proliferation and apoptosis, and in the invasion and migration of BC MDA-MB-231, MDA-MB-468, and MCF-10AT cells (Figure 2).

#### 2.1.4 Cervical Cancer

Cervical cancer (Moore, 2006; Li et al., 2019; Gao et al., 2020) is one of the most common gynecological cancers in women worldwide. Infection with human papilloma virus (Burd, 2003; Olusola et al., 2019) is the leading pathogenic risk factor for development of cervical cancer. Newly developed molecular technologies have been used to reveal a variety of new molecular characteristics for clinical therapies (The Cancer Genome Atlas Research Network, 2017). LINC00707 is markedly overexpressed in 20 cervical cancer tissues and cell lines (e.g., HCC94, CaSki, MS751, HT-3, and C-33A) (Guo et al., 2021). Xenograft mouse models have revealed that LINC00707 overexpression is strongly linked to increased tumor volumes and weights. LINC00707 exerts its oncogenic functions to prompt cervical cancer development by modulating biological processes such as cell proliferation, migration, and invasion in C-33A and HT-3 cells.

#### 2.1.5 Bladder Cancer

Bladder cancer (Dobruch et al., 2016; Aghaalikhani et al., 2019; Cao et al., 2021) is the most frequent urinary system malignancy. LINC00707 expression is markedly upregulated in bladder cancer (Gao and Ji, 2021) tissues from 103 patients at the Weifang People’s Hospital and UMUC3 and T24T cells. Overexpression of LINC00707 is associated with tumor stage, grade, poor overall survival, and disease-free survival. Biological functional studies have found that LINC00707 is involved in the induction of cell proliferation, colony formation, and metastasis of UMUC3 and T24T cells.

#### 2.1.6 Gastric Cancer

Gastric cancer (Jemal et al., 2011; Yasunaga et al., 2013) is the most common malignancy of the human digestive system. Findings of mechanism studies highlight that LINC00707 has emerged as a crucial promotor in gastric cancer initiation and development. LINC00707 is markedly expressed in 60 gastric cancer patients’ tissues (Xie et al., 2019) and cell lines. Upregulation of LINC00707 is clinically linked to increased TNM stage, tumor size, lymphatic metastasis, and an unfavorable prognosis in patients with gastric cancer. LINC00707 also has pivotal oncogenic functions in the processes of cell proliferation and metastasis in BGC-823 and SGC-7901 cells.

#### 2.1.7 Glioma

LINC00707 is overexpressed in glioma tissues (Liu and Hu, 2020; Yu et al., 2021) and cells. This high expression is associated with poor glioma clinicopathological parameters, including Karnofsky performance status score and WHO grade (Liu and Hu, 2020). LINC00707 enables modulation of diverse biological behaviors in gliomas with respect to cell proliferation, invasion, and migration, and together with generation of VM of U87 and U251 cells. (Liu and Hu, 2020; Yu et al., 2021).

#### 2.1.8 Colorectal Cancer

As an oncogene, LINC00707 participates in the modulation of CRC (Shao et al., 2019; Zhu H. et al., 2019; Wang et al., 2020). High levels of LINC00707 expression occur in both CRC tissues and cell lines and are strongly associated with large tumor size (Zhu H. et al., 2019; Wang et al., 2020), advanced tumor stage, lymphatic and distant metastases, and poor survival. LINC00707 exerts cancer-promoting activity, as it participates in cell proliferation, invasion, and migration of LoVo, HT29, SW480, SW620, and HCT116 cells.

#### 2.1.9 Hepatocellular Carcinoma

Noncoding RNAs are implicated in carcinogenesis, including of HCC (Bruix and Sherman, 2011; Galuppo et al., 2014). LINC00707 is highly expressed in HCC cell lines (e.g., HepG2, Huh7, Hep3B, and SNU449) and tissues. It can regulate the cell cycle, enhance cell proliferation, migration, and invasion capacity, and repress cell apoptosis of HepG2, Huh7, Hep3B, and SNU449 cells.

#### 2.1.10 Ovarian Cancer

A study of biomarkers for ovarian cancer in patients exposed to bisphenol A (Zahra et al., 2021) found that LINC00707 levels are significantly increased in ovarian cancer tumor tissues compared with adjoining healthy tissues. Using the SKOV3 cell exposed to bisphenol A, LINC00707 has been found to associate with poor overall survival.

#### 2.1.11 Melanoma

Expression of LINC00707 is distinctly higher during early-stage melanoma (Yang et al., 2018). More importantly, LINC00707, which is contained within the six-lncRNA signature, may have potential roles in melanoma risk-stratification and survival prognosis via signaling pathway types such as the mitogen-activated protein kinase (MAPK) pathway, immune and inflammation-related pathways, the neurotrophin signaling pathway, and focal adhesion pathways.

#### 2.1.12 Clear Cell Renal Cell Carcinoma

LINC00707 is overexpressed in 526 clear cell renal cell carcinoma tissues (Pang et al., 2020) and cell lines. It promotes proliferative, migratory, and invasive biological processes of 786-O and 769-P cells via the epithelium-to-mesenchymal transition pathway.

### 2.2 Other Diseases

#### 2.2.1 Pneumonia

Pneumonia (Liu et al., 2018; Schicke et al., 2020) is the most frequent and fatal infectious disease of the lower respiratory tract worldwide. It typically manifests as a series of lung inflammatory responses. Precise identification of the underlying associated pathogens to implement effective anti-infective therapy remains a challenge (Meyer Sauteur, 2020). Sensitive diagnostic tools to guide pneumonia treatment (Naydenova et al., 2016) are required. LINC00707 (Zou et al., 2021) is highly expressed in damage caused by lipopolysaccharide (LPS) in MRC-5 cells. LINC00707 participates in biological functions mediated by LPS, such as cell viability, apoptosis, and inflammatory status in MRC-5 cells.

#### 2.2.2 Spinal Cord Injury

LINC00707 is a vital regulator involved in the progression of SCI (Zhu S. et al., 2019). Its expression is significantly increased in SCI LPS-treated PC-12 cells, where it consequently exerts pro-apoptotic and pro-inflammatory effects on LPS-treated cells by binding to miR-30a-5p and inhibiting Neurod 1 expression. These results may reveal a new strategy for clinical treatment of SCI.

## 3 Mechanisms of LINC00707 in Disease

LINC00707 participates in the regulation of a diverse range of biologic functions, including tumor cell proliferation, apoptosis, invasion, metastasis, cell cycle regulation, and osteogenesis. The following sections mainly focus on the molecular mechanisms of LINC00707 in regulating biological functions of diseases.

### 3.1 Cell Proliferation

Dysregulation of the cell cycle leads to unlimited cell proliferation and further tumor formation (Tu et al., 2020). LINC00707 has a pro-oncogenic role and promotes cell proliferation in cancers such as LUAD (Ma et al., 2018), osteosarcoma, BC (Yuan et al., 2020), CC (Guo et al., 2021), bladder cancer (Gao and Ji, 2021), glioma (Liu and Hu, 2020), HCC (Tu et al., 2019; Wang et al., 2019), and clear cell renal cell carcinoma (Pang et al., 2020).

In LUAD SPCA1 and A549 cells, LINC00707 fosters cell proliferation via direct enhancement of expression of target gene cell division cycle 42 (Cdc42) (Ma et al., 2018). Experiments on osteosarcoma MG-63 and Saos-2 cells revealed that LINC00707 exploits positive regulatory effects on cell proliferation by acting as a competing endogenous RNA (ceRNA) of miR-338-3p and further heightening AHSA1 expression (Zhang et al., 2021). In BC (Yuan et al., 2020), LINC00707 sponges miR-30c to affect CTHRC1 expression, thus enhancing the proliferative ability of BC MDA-MB-231 and MDA-MB-468 cells (Yuan et al., 2020). LINC00707 can also interact with miR-206 to regulate expression of target protein ER-α, which affects proliferation of precancerous breast MCF-10AT cells (Jiang et al., 2020). Likewise, in cervical cancer HT-3 (Guo et al., 2021) and C-33A cells, LINC00707 functions as an miRNA sponge to restrain levels of miR-382-5p and elevate expression of VEGFA to result in more intense cell proliferation. In bladder cancer (Gao and Ji, 2021), LINC00707 is involved in Wnt/β-catenin signaling to contribute to the proliferation of UMUC3 and T24T cells, through sponging miR-145 to modulate CDCA3 expression. In GC (Xie et al., 2019), LINC00707 can regulate the stability of its downstream target VAV3/F11R mRNAs to promote proliferation of GC BGC-823 and SGC-7901 cells by interacting with the mRNA stabilizing protein HuR. In glioma (Yu et al., 2021), LINC00707 directly binds miR-613/miR-651-3p, and thus promotes proliferation of U87 and U251 cells. In CRC, LINC00707 accelerates cell proliferation, and even the cell cycle from the G1 to S phase, via sponging miR-206 to increase expression of target proteins FMNL2 (Shao et al., 2019) in SW620, and HCT 116 cells, NOTCH3 (Zhu H. et al., 2019) and TM4SF1 in LoVo and HCT116 cells, or via binding with miR-485-5p (Wang et al., 2020) in HT29 and HCT116 cells. In HCC (Tu et al., 2019; Wang et al., 2019), LINC00707 knockdown inhibits HCC cell proliferation, cell cycle progression, and colony formation via depression of the ERK/JNK/AKT signaling pathway in Hep3B and SNU449 cells or via regulation of miR-206 and CDK14 *in vivo* and in HepG2 and Huh7 cells.

### 3.2 Cell Apoptosis

**FIGURE 3:**
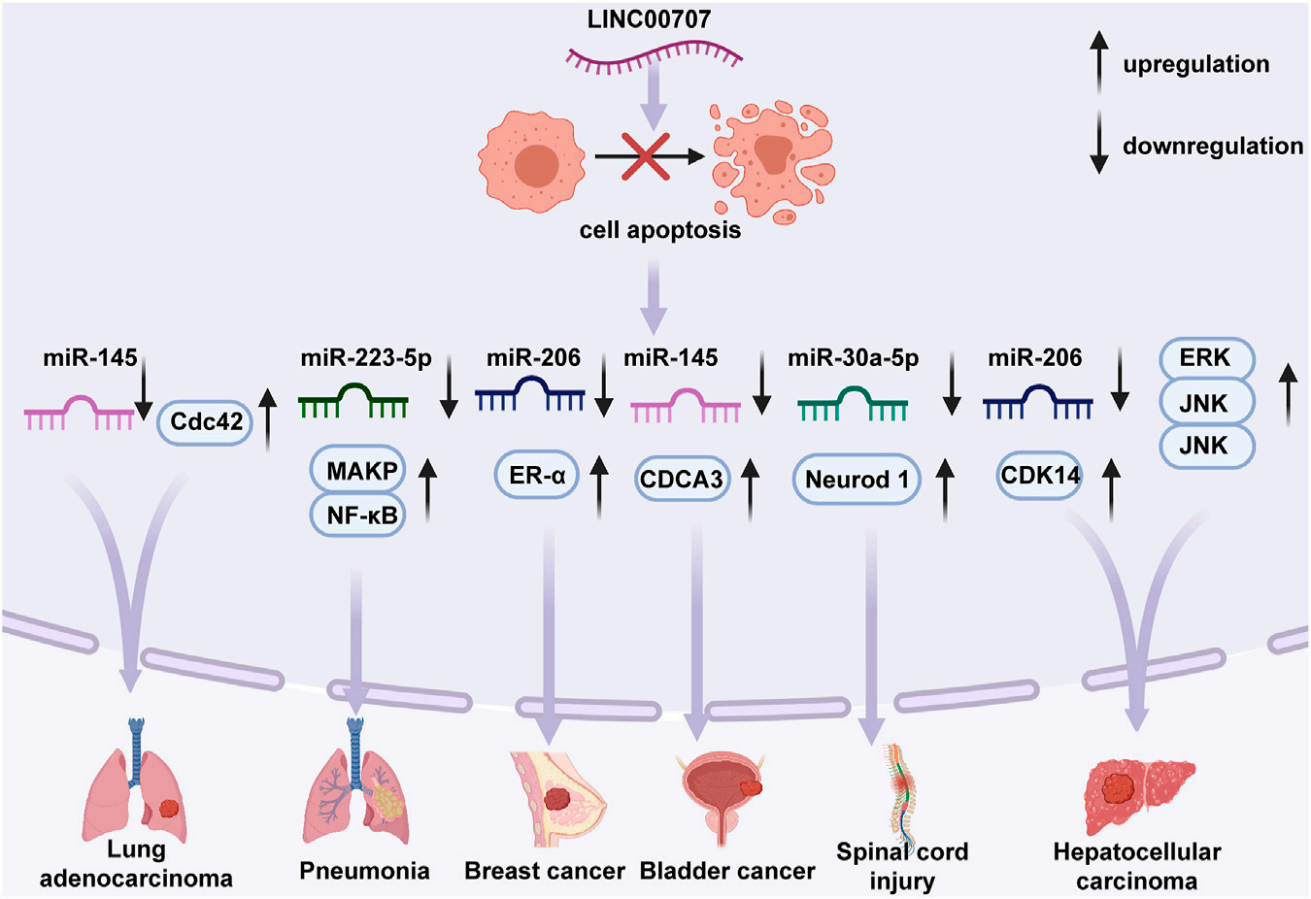
Regulative functions of LINC00707 in the apoptotic mechanisms of certain cell types. In lung adenocarcinoma, LINC00707 suppresses cell apoptosis by activating expression of Cdc42 or inhibiting expression of miR-145. In pneumonia, LINC00707 binds to miR-223-5p and regulates p38 mitogen-activated protein kinase (MAPK), and nuclear factor-κB (NF-κB) signaling pathways. In breast cancer, LINC00707 suppresses miR-206 expression and upregulates ESR1 expression. In bladder cancer, LINC00707 combines with miR-145 and affects expression of CDCA3. In hepatocellular carcinoma, LINC00707 mediates activity of the ERK/JNK/AKT pathway or interacts with miR-206 to regulate expression of CDK14. In spinal cord injury, LINC00707 binds to miR-30a-5p and inhibits expression of Neurod 1.

Cell apoptosis (Liu et al., 2009; Yamanouchi et al., 2010; Seyrek et al., 2019) is the common process of programmed cell death. Abnormal regulation of apoptosis has an important role in cancer pathogenesis. In LUAD (Ma et al., 2018), LINC00707 suppresses cell apoptosis by activating Cdc42 expression. LINC00707 directly inhibits miR-145 expression to reduce NSCLC A549 cell apoptosis (Zhang et al., 2019). Large studies of patients with pneumonia found that LINC00707 binds to miR-223-5p, and subsequently regulates p38 MAPK and nuclear factor-κB (NF-κB) signaling pathways to aggravate LPS-mediated MRC-5 cell apoptosis (Zou et al., 2021). In BC, LINC00707 is hypothesized to be involved in inhibition of MCF-10AT cell apoptosis (Jiang et al., 2020) and to drive the cell cycle from the G1 to S and the G2 phase via blocking miR-206 expression and upregulating ESR1 expression. LINC00707 serves as a ceRNA to combine with miR-145 and thus affect CDCA3 expression and decrease the apoptotic cell number of bladder cancer (Gao and Ji, 2021) UMUC3 and T24T cells. In HCC (Tu et al., 2019; Wang et al., 2019), the antiapoptotic effects of LINC00707 are via mediation of the activity of the ERK/JNK/AKT pathway in Hep3B and SNU449 cells or via interaction with miR-206 to regulate CDK14 expression in HepG2 and Huh7 cell (Figure 3).

### 3.3 Cell Migration and Invasion

Invasion and migration are the major causes behind the high mortality rate of diverse types of cancer (Liu L. et al., 2020) and high rates are characteristic of advanced malignancies. In LUAD, LINC00707 overexpression can induce cell migration and invasion via triggering Cdc42 expression (Ma et al., 2018). In osteosarcoma, LINC00707 binds to miR-338-3p and increases AHSA1 expression to promote cell migration and invasion (Zhang et al., 2021). In BC MDA-MB-231 cells and MDA-MB-468 cells, LINC00707 acts as a miRNA sponge to interact with miR-30c and actively stimulate CTHRC1 expression; it thus promotes BC cell invasion and migration (Yuan et al., 2020). CC mechanism studies revealed that LINC00707 can combine with miR-331-3p to regulate expression of target VEGFA, and thus contribute to cervical cancer (Guo et al., 2021) HT-3 and C-33A cell migration and invasion.

*In vitro* bladder cancer assays (Gao and Ji, 2021) revealed LINC00707 knockdown leads to damaged premetastatic abilities of UMUC3 and T24T cells via targeting miR-145 and further inhibition of CDCA3 expression. In GC (Xie et al., 2019), LINC00707 combines with HuR, thereby reinforcing the stability of the mRNA targets VAV3 and F11R and promoting BGC-823 and SGC-7901 tumor cell metastasis. In glioma (Liu and Hu, 2020; Yu et al., 2021), LINC00707 enhances migration and invasion capacities of glioma U87 and U251 cells via direct binding of LINC00707 to miR-613 or miR-651-3p. In CRC, LINC00707 modulates protein expression of FMNL2 (Shao et al., 2019), NOTCH3 (Zhu H. et al., 2019), and TM4SF1 by sponging miR-206 and thus making LoVo, SW620, and HCT 116 cells more aggressive. During HCC migration and invasion (Tu et al., 2019; Wang et al., 2019), LINC00707 induces cell migration and invasion of HepG2, Huh7, Hep3B, and SNU449 cells by downregulating miR-206 to increase CDK14 levels or via deactivation of the ERK/JNK/AKT pathway.

### 3.4 *In Vivo* Studies

A LUAD xenograft tumor model that found smaller tumor sizes, lower tumor weights, and slower tumor growth after LINC00707 knockdown (Ma et al., 2018) further supported the tumor-promoting properties of LINC00707. In a GC nude mouse model (Xie et al., 2019), the phenomenon of larger tumor volumes and weight, a faster tumor growth and epithelium-to-mesenchymal transition process, and fewer metastatic nodules further supported the hypothesis that LINC00707 can promote GC cell tumorigenesis and metastasis. A xenograft experiment using glioma nude mice (Yu et al., 2021) found that LINC00707 knockdown attenuates the process of tumor growth, results in a better survival rate and less VM formation, and revealed the value of the protumor for the occurrence and development of glioma. A tumor xenograft experiment that was performed in mice with cervical cancer (Guo et al., 2021) revealed reduced tumor volumes and tumor weights when LINC00707 was lacking. These results supported the hypothesis that LINC00707 has a role in cervical cancer growth. A CRC *in vivo* xenograft nude mice model (Shao et al., 2019) revealed that tumors grow more slowly under conditions of LINC00707 knockdown and further support the cancer-promoting ability of LINC00707. A BALB/c mouse animal study of HCC (Tu et al., 2019) also found that downregulation of LINC00707 hinders HCC carcinogenesis.

## 4 LINC00707 in Clinical Settings

### 4.1 LINC00707 as a Diagnosis and Prognosis Biomarker

Study findings support the hypothesis that abnormal LINC00707 expression in cancer tissues is a useful marker for the clinical diagnosis and prognosis of cancers including lung adenocarcinoma (Ma et al., 2018), BC (Jiang et al., 2020; Yuan et al., 2020), bladder cancer (Gao and Ji, 2021), melanoma (Yang et al., 2018), GC (Xie et al., 2019), and CRC (Wang et al., 2020). The differential expression of LINC00707 in normal and pathological tissues and cell lines has made it the hopeful and powerful tool of choice for disease diagnosis. However, the expression of LINC00707 in some types of disease and specific physiological processes remains unclear, such as the process of osteogenic differentiation. Considering the limited research recently, further studies are necessary to confirm the exact expression in diseases. In addition, there is little evidence concerning the stability and distribution of LINC00707 in human plasma, serum, urine and other body fluids at present, hindering the LINC00707 application in diagnosis. Blood and urine are easily available body fluids which are capable of reflecting the systemic metabolic activity. Further studies for the expression, sensitivity and stability of LINC00707 in non-invasive body fluids are required to make LINC00707 an ideal tool for disease diagnosis.

A study of LUAD in hypoxic conditions found that a constructed seven-lncRNA prognostic model that contained LINC00707 had reliable predictive ability, based on area under the receiver operating characteristic curve results (Shao et al., 2021). LINC00707 overexpression to reduces disease-free and overall survival (Ma et al., 2018). In hypoxic conditions, LINC00707 also mirrors the tumor immunological homeostatic response; its use improves the prognosis (Ma et al., 2018; Shao et al., 2021) of patients with LUAD.

### 4.2 LINC00707 as a Disease Treatment Target

LINC00707 is widely engaged in the processes of cell proliferation, apoptosis, and metastasis for a range of diseases, including LUAD (Ma et al., 2018; Zou et al., 2021), osteosarcoma (Jiang et al., 2020; Zhang et al., 2021), BC (Yuan et al., 2020), glioma (Liu and Hu, 2020; Yu et al., 2021), CRC (Shao et al., 2019; Zhu H. et al., 2019), and pneumonia (Zou et al., 2021). The differential expression of LINC00707 in a broad range of diseases and physiological processes can also provide a new insight into drug candidate for the disease treatment. We can employ the pro-oncogenic role of LINC00707 in some disease to achieve a therapeutic effect by suppressing its expression. And the application of the anti-oncogenic role in other disease can also attain the therapeutic impact via enhancing its level. On the other hand, LINC00707 knock-down or agonist/antagonist addition based on its molecular mechanisms in disease may act as essential druggable targets for the development of new therapies. For example, LINC00707 knockdown enables relief of LPS-induced cell injury and inflammation, which is a potential novel therapeutic approach for patients with pneumonia (Zou et al., 2021). A study of BC revealed that acetyl-11-keto-β-boswellic acid (Jiang et al., 2020) may have therapeutic effects on precancerous breast lesions via the LINC00707/miR-206 axis, and therefore mediate the ER-α protein of ESR1 expression to inhibit the estrogen signaling pathway. Remarkably, one study found that LINC00707 involvement is necessary for the regulation of VM formation via the HNRNPD/ZHX2/LINC00707/miR-651-3p/SP2 axis. These results suggest a novel approach for anti-VM therapy for glioma (Yu et al., 2021). It is also found to intensify the DDP IC50 value and consequent involvement in the progression of cisplatin (DDP) resistance in NSCLC A549 cells (Zhang et al., 2019). However, current information regarding LINC00707 for treatment application is limited, the efficacy, stability and safety of LINC00707-targeted drugs need further pre-clinical and clinical studies.

Taken together, these findings all suggest that LINC00707 is a promising treatment target for a variety of diseases. Further studies for the expression, sensitivity, and stability of LINC00707 in non-invasive body fluids for disease diagnosis in addition to the efficacy, and safety of LINC00707-targeted drugs for disease treatment was required.

## 5 Conclusion

LINC00707 is now widely accepted to have critical functions in the regulation of diverse pathological and biological processes, together with tumor development and progression through specific pathways. Recently, an increasing number of studies has revealed that LINC00707 is a dysregulated lncRNA in multiple types of diseases. Results indicate it has considerable involvement in a variety of complex clinicopathological characteristics, such as advanced tumor TNM stage, larger tumor size, lymphatic metastasis, distant metastasis, and a shorter overall survival time.

LINC00707 widely participates in various cellular processes, including cellular proliferation, apoptosis, and migration in most types of tumors, and in the process of osteogenic differentiation and the inflammatory conditions induced by LPS. Results of studies on mechanistic pathways suggest that LINC00707 mainly exerts its biological function by binding with relevant miRNA and consequently regulating expression of downstream targets, including miR-338-3p/AHSA, miR-382-5p/VEGFA, miR-145/CDCA3, miR-30c/CTHRC1, miR-103a-3p/DKK1, and miR-370-3p/WNT2B. Taken together, results of studies of LINC00707 could provide us with breakthroughs for the diagnosis, prognosis, and treatment of various diseases, including cancers. Given that LINC00707 research is currently in its nascent stages and studies have not fully revealed its functions, in-depth studies are needed to further elucidate the regulatory mechanisms of LINC00707 in numerous diseases.
